# ToxTracker Reporter Cell Lines as a Tool for Mechanism-Based (Geno)Toxicity Screening of Nanoparticles—Metals, Oxides and Quantum Dots

**DOI:** 10.3390/nano10010110

**Published:** 2020-01-06

**Authors:** Sarah McCarrick, Francesca Cappellini, Amanda Kessler, Nynke Moelijker, Remco Derr, Jonas Hedberg, Susanna Wold, Eva Blomberg, Inger Odnevall Wallinder, Giel Hendriks, Hanna L. Karlsson

**Affiliations:** 1 Institute of Environmental Medicine, Karolinska Institutet, 171 77 Stockholm, Sweden; 2 KTH Royal Institute of Technology, Division of Surface and Corrosion Science, Department of Chemistry, 100 44 Stockholm, Sweden; 3 Toxys, 2333 CG Leiden, The Netherlands; 4 Division Bioscience and Materials, RISE Research Institutes of Sweden, 111 21 Stockholm, Sweden

**Keywords:** nanotoxicology, genotoxicity, DNA damage, metal oxides, high throughput screening

## Abstract

The increased use of nanoparticles (NPs) requires efficient testing of their potential toxic effects. A promising approach is to use reporter cell lines to quickly assess the activation of cellular stress response pathways. This study aimed to use the ToxTracker reporter cell lines to investigate (geno)toxicity of various metal- or metal oxide NPs and draw general conclusions on NP-induced effects, in combination with our previous findings. The NPs tested in this study (*n* = 18) also included quantum dots (QDs) in different sizes. The results showed a large variation in cytotoxicity of the NPs tested. Furthermore, whereas many induced oxidative stress only few activated reporters related to DNA damage. NPs of manganese (Mn and Mn_3_O_4_) induced the most remarkable ToxTracker response with activation of reporters for oxidative stress, DNA damage, protein unfolding and p53-related stress. The QDs (CdTe) were highly toxic showing clearly size-dependent effects and calculations suggest surface area as the most relevant dose metric. Of all NPs investigated in this and previous studies the following induce the DNA damage reporter; CuO, Co, CoO, CdTe QDs, Mn, Mn_3_O_4_, V_2_O_5_, and welding NPs. We suggest that these NPs are of particular concern when considering genotoxicity induced by metal- and metal oxide NPs.

## 1. Introduction

The use of nanoparticles (NPs) is increasing worldwide in various applications, resulting in a need to more rapidly evaluate their potential toxicity. The genotoxic potential is a crucial part in the risk and safety evaluation of NPs and currently this is most often evaluated by a battery of established methods including the comet assay and the micronucleus assay [1,2,3]. However, these methods are relatively time-consuming and there is consequently a great demand for efficient and more high throughput assays for screening of the genotoxicity of NPs [4]. As reviewed by Nelson et al. [5], some of the established genotoxicity assays have been modified and optimized to give increased sample capacity and throughput such as flow cytometry based micronucleus assay as well as comet on a chip.

An alternative approach with great potential is the use of reporter cell lines, which are designed to fluorescence upon activation of specific signaling pathways. In the field of genotoxicity there are some validated reporter assays available such as the GreenScreen HC assay [6] and the luminescence-based reporter assay Bluescreen HC [7]. Both assays target GADD45α, which is induced upon various cellular stresses including genotoxic stress. A combination of several reporter cell lines is required to provide a more mechanistic evaluation of the genotoxic potential. The ToxTracker reporter assay consists of a panel of six mouse embryonic stem (mES) cell lines that are modified with different green fluorescent protein (GFP) tagged reporters for various cellular signaling pathways involved in carcinogenesis [8,9]. By using this set of reporter cell lines, it is possible to monitor the activation of signaling pathways associated with DNA damage, oxidative stress, general p53-dependent cellular stress as well as protein unfolding response (see Table 1). We have previously shown the ToxTracker assay to be a valid reporter assay for detecting (geno)toxic properties of NPs with results correlating to those of more conventional assays [10]. Furthermore, we have shown the applicability of these reporters for various NPs including nickel-based [11], cobalt-based [12], and welding fume NPs [13].

In the present study we focus on a variety of metal-containing NPs including quantum dots (QDs). Metal based NPs are industrially relevant nanomaterials being widely used in consumer products such as cosmetics, food products, batteries as well as in medicinal applications [14,15]. Metal oxide NPs often have semi-conductive and catalytical properties and are therefore appealing from a technical point of view and often produced in large scale at industrial settings. Therefore, the potential occupational and non-occupational exposure to metal NPs is increasing.

Quantum dots (QDs) are fluorescent semiconducting nanocrystals that, due to their small size, have distinctive optical and electronic properties resulting in bright and highly stable fluorescence. Their small sizes in combination with large specific surface areas make QDs able to target ligands for site-directed activity [16,17]. Absorption and emission of QDs are dependent on properties such as composition, crystal structure and size, which result in unique physicochemical properties for each individual type of QDs [18]. QDs have for this reason been proposed to not be considered as a uniform group of nanomaterials [16]. Cadmium-containing QDs, including cadmium telluride (CdTe) and cadmium selenide, are among the most commercially available types and considered particularly attractive for optical, bioanalytical and bio imaging applications where CdTe utilizes the infrared regions [17,18,19]. CdTe QDs have been shown to induce various toxic effects in vitro including cytotoxicity and generation of reactive oxygen species (ROS) [20,21].

Although a large number of studies have investigated the in vitro genotoxicity of e.g., various metal oxides, as reviewed in Golbamaki et al. [22] and Magdolenova et al. [3], the underlying mechanisms are still not clear. Furthermore, few studies have focused on comparing metal NPs and corresponding oxides as well as the importance of size for their genotoxic potency. The aim of this study was to use the ToxTracker reporter cell lines to investigate (geno)toxicity of various metal- or metal oxide NPs as well as QDs of different size. A further aim was to summarize and draw general conclusions on toxicity of NPs as well as underlying pathways using our present and previous work with the ToxTracker reporter cell lines.

## 2. Materials and Methods

### 2.1. Nanoparticles

The CdTe QDs (1.5–8.6 nm) were purchased from PlasmaChem GmbH (Berlin, Germany). Mn (20–40 nm), Mn_3_O_4_ (<100 nm), Cr (35–45 nm), Cr_2_O_3_ (10–30 nm), Sn (10–20 nm) and SnO_2_ (20–40 nm) NPs were all obtained from American Elements (Los Angeles, CA, USA). V (80–100 nm) and V_2_O_5_ (80 nm) NPs were purchased from Nanoshel (Wilmington, DE, USA), Sb NPs (N/A) from Campine NV (Beerse, Belgium) and Sb_2_O_3_ NPs (150 nm) from Sigma Aldrich (Ramstadt, Germany). Au, Ag and Pt NPs (5 nm) were obtained from nanoComposix (San Diego, CA, USA) in the form of stock dispersions (1 mg/mL) in aqueous 2 mM citrate (Ag and Pt) and in ultrapure water (milli-Q) (Au).

For the NPs in dry powder form, NP suspensions of 1 mg/mL in cell medium were sonicated for 2 × 10 min in an ultrasonic water bath alternated by 10 s vortexing. The particle suspensions were freshly prepared for every experiment and further diluted to desired concentrations in cell media immediately before the cell exposure.

### 2.2. Particle Morphology

Transmission electron microscopy (TEM) was employed to study the primary size and shape of the NPs received as powders. Three drops of a particle suspension of 1 mg/mL ethanol were applied to 200 mesh TEM copper grids with formvar/carbon support films (Ted Pella, Inc., Redding, CA, USA). The suspension was sonicated for 15 min and vortexed before applied onto the grid. Drying was made at ambient laboratory conditions. Imaging was conducted using a HT7700 TEM (Hitachi, Tokyo, Japan) instrument operating at 100 kV.

TEM images of the Au, Ag and Pt NPs are reported elsewhere [23]. No TEM imaging was made on the CdTe QDs.

### 2.3. Characterization of Particle Agglomeration

PCCS (Photon cross correlation spectroscopy) was used to study changes in size distribution of the particles, agglomeration and concentration (possible reduction due to sedimentation) over time in the cell medium. Particle suspensions of 1 mg/mL medium were prepared by ultrasonication for 2 × 10 min alternated by 10 s vortexing at 37 °C. The samples were prepared in disposable single sealed cuvettes, LOTG17501P (Eppendorf AG, Hamburg, Germany) at a concentration of 0.1 mg/mL. The samples were stored at 37 °C and measured in triplicates after 0, 2 and 24 h using the NANOPHOX 90–250 V (Sympatec GmbH, Claustahl-Zellerfeld, Germany) instrument. Windox 5, the PCCS software (version 10, SympatecGmbH, Claustahl-Zellerfeld, Germany), was used to fit the size distribution data for each measurement. Measurements of standard latex particles (100 nm) and non-particle containing media were performed to ensure high quality data. The samples were measured after 24 h. Measurements were performed on the metal and metal oxide NPs as well as the QDs.

Particle size measurements of the Au, Ag and Pt NPs were determined using NTA (Nanoparticle Tracking Analysis) as described in Lebedova et al. [23].

### 2.4. Cell Culture and Reagents

mES cells were cultured as previously described [9]. In short, the cells were maintained in the presence of irradiated mouse embryonic fibroblasts as feeder cells in knockout DMEM supplemented with 10% FBS, 2 mM GlutaMAX, 1 mM sodium pyruvate, 100 mM b-mercaptoethanol and leukemia inhibitory factor (LIF). Prior to exposure, cells were seeded at a density of 4 × 10^4^ cells per well on gelatin-coated 96-well plates in complete mES cell medium in the absence of feeder cells for 24 h.

### 2.5. ToxTracker Assay (mES Cells)

An initial screening of the cytotoxicity of the NPs was performed in non-modified mES cells at increasing concentrations of the NPs (0–100 µg/mL) at 24 h. Based on this, the doses for GFP reporter analysis were determined so that the highest dose caused 50–75% cytotoxicity. Four additional doses in two-fold dilution steps from the highest dose were then selected. In cases of no observed cytotoxicity, the NPs were tested up to 100 µg/mL. Thus, some of the NPs were not tested up to cytotoxic concentrations. Following exposure, cells were washed and trypsinized. Induction of GFP was determined using a Guava easyCyte 8HT flow cytometer (Millipore, Burlington, MA, USA) in intact single cells as described previously [9]. Mean GFP fluorescence was used to calculate GFP reporter induction compared to vehicle control exposure. Cytotoxicity was assessed by cell count after 24 h exposure using flow cytometry and expressed as percent of intact cells compared to vehicle control exposure. Possible autofluorescence was recorded for the NPs tested, and in the case of CdTe QDs subtracted for in the GFP-reporter values. Presented results are based on at least three independent experiments and error bars represent standard error of the mean. The GFP response is considered positive if exceeding a 2-fold increase, which is based on previous validations [8,9]. All the GFP-reporters were tested with the positive controls cisplatin (2.5–10 µM), diethyl maleate (62.5–250 µM) and tunicamycin (1–4 µM). Similar as to what was reported by Hendriks et al. [8], cisplatin resulted in an induction of the reporters Bscl2, Rtkn, Btg2, Srxn1 and Blvrb, diethyl maleate induced both of the oxidative stress reporters Srxn1 and Blvrb while tunicamycin induced solely the Ddit3 GFP-reporter.

### 2.6. Dose Metric Modelling Analysis

Firstly, the number of particles per unit of volume was calculated for each concentration used, based on information from the supplier. The dose-response data for all reporter cells was analyzed with the PROAST software (version 38.9, National Institute for Public Health and the Environment (RIVM), Bilthoven, the Netherlands) to find the number of QDs needed to induce a 20% decrease in viability. The relationship among these equi-response doses was used to identify the appropriate dose metric as described in Delmaar et al. [24]. Briefly, the equi-response doses were plotted as a point in the plane spanned by the 10log of the diameter and the 10log of the number of particles. If the administered surface area or volume or number of particles is the appropriate metric, then the resulting curve should be a straight line, mathematically described as:

log(*N*) = m × log(*d*) + q (1)

where *N* is the number of particles and *d* is the diameter of the NPs. The slope of this curve describes the correct metric: surface area has a slope equal to −2, volume equal to −3 and number of particles equal to 0. If the slope is equal to none of these numbers, then the dose metric is:

10^q^ = *N* × *d*^−m^ (2)

### 2.7. Statistical Analysis

Results are expressed as mean values of at least three independent experiments (*n* = 3) ± standard error of the mean (SEM), except for PCCS data expressed as mean values ± standard deviation. LD50 (lethal dose in 50%) values were estimated by dose-response modeling assuming Hills slope (slope = 1). LD50 values as well as linear regression for dosimetry were calculated using GraphPad Prism 5.02 statistical software (GraphPad Inc., La Jolla, CA, USA).

## 3. Results

### 3.1. Particle Characterization

**Figure 1:**
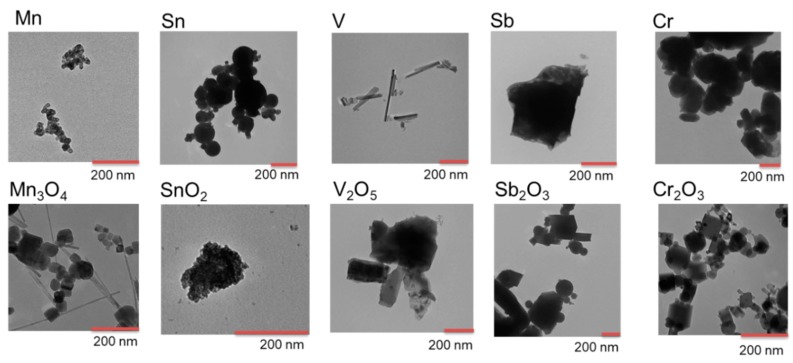
TEM images of the metal and metal oxide NPs.

TEM observations revealed a majority of the metal and metal oxide particles to be approximately spherical with primary sizes ranging from 10 to 250 nm, Figure 1 and Table 2. Main outliers in primary size were observed for NPs of Mn_3_O_4_, V, V_2_O_5_, Sb and SnO_2_. The Mn_3_O_4_ NPs comprised smaller cubic particles (similar to observations for the other NPs) and long rods with a length scale up to 8 µm. Rods (nm-sized) were the predominating shape for the V NPs. The V_2_O_5_ and the Sb NPs had irregular shapes and primary particle sizes up to 1.2 µm. The SnO_2_ NPs formed aggregates of individual particles sized 2–3 nm. Compositional information of the outermost surface (oxide particles) and surface oxide (metal particles) of the NPs is given in Table 2 based on XRD, Raman, and XPS findings based on [25,26,27,28,29,30,31,32,33,34] (see supporting information, Figures S1–S3). The main surface oxides observed for the metallic NPs were in general similar to observations for the corresponding bulk metal oxide with the exception for the Mn NPs (Table 2).

The primary size of Au, Ag and Pt NPs were previously confirmed to be 5 nm [23]. Primary particle sizes of the CdTe QDs are reported in Table 2 based on supplier information. No compositional analyses were performed.

**Figure 2:**
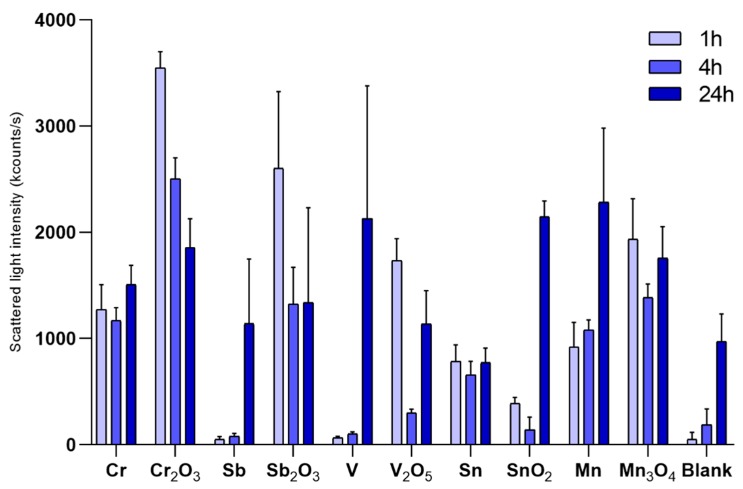
Changes in scattered light intensities for the different metal and metal oxide NPs after 0, 2, and 24 h of exposure in cell medium recorded by means of PCCS. Particle loading: 100 µg/mL; Blank cell medium only without any NPs. The error bars represent the standard deviation based on three independent samples.

Changes in scattered light intensities of the NPs over time in the ToxTracker cell medium is presented in Figure 2 together with parallel measurements for the blank solution (cell medium without NPs). Agglomeration of cell medium constituents (no NPs) with time was evident (increased light intensities with time). Observations with similar count rates between blank solutions (no NPs) and the NP-containing solutions indicate NP sedimentation. If no changes in intensities are observed with time and sedimentation of the NPs has taken place, measured count rates relate to agglomerated medium constituents. Low count rates observed already at time 0 h imply rapid sedimentation taking place before any measurements have been made (<5 min) (i.e., during dispersion preparation as indicated by low particle concentration in solution).

Most NPs showed evident signs of sedimentation over time (most notable after 24 h), except for the Mn NPs and to some extent the SnO_2_ NPs (reduced intensities or similar intensities with time compared to findings for the blank solution). The Mn NPs seemed to have a higher ability to remain dispersed in solution over time (increased intensities with prolonged exposure). The SnO_2_ NPs showed signs of sedimentation already at time 0 h and after 2 h (reduced light intensity), but increased intensities after 24 h. This effect could possibly be related to agglomeration of cell medium constituents. Almost complete sedimentation was observed for Sb and V NPs immediately upon sample preparation (similar intensities as the blank at time 0 h).

As a result of extensive particle sedimentation due to formation of large agglomerates, any estimate of particle size distributions in solution become very approximate and hence not reported.

Sizes of the QDs were not possible to discern by means of PCCS in the cell media due to their small size and similar size range as the cell media components only. Time dependent measurements implied no evident agglomeration of the QDs up to 24 h.

### 3.2. Nontoxic NPs—Several NPs Cause No Toxicity or ToxTracker Activation

**Figure 3:**
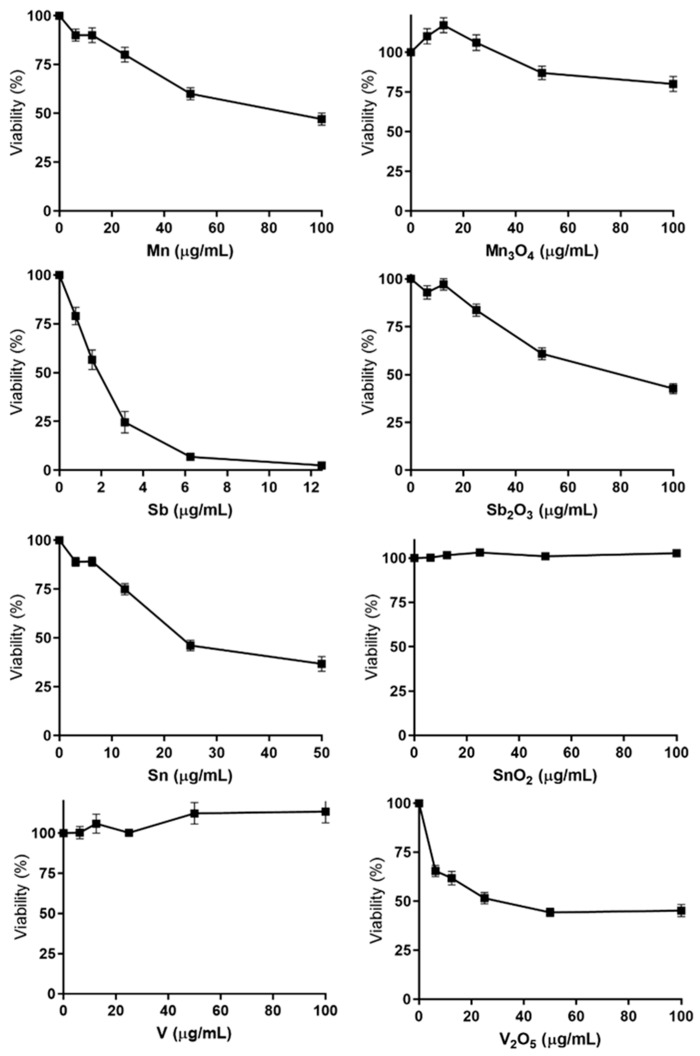
Cytotoxicity in mES cells following 24 h exposure to metal and metal oxide NPs. Cytotoxicity was determined by measuring the fraction of intact cells following exposure using flow cytometry. The results are presented as mean ± standard error of the mean of three or four independent experiments (*n* = 3–4).

**Figure 4:**
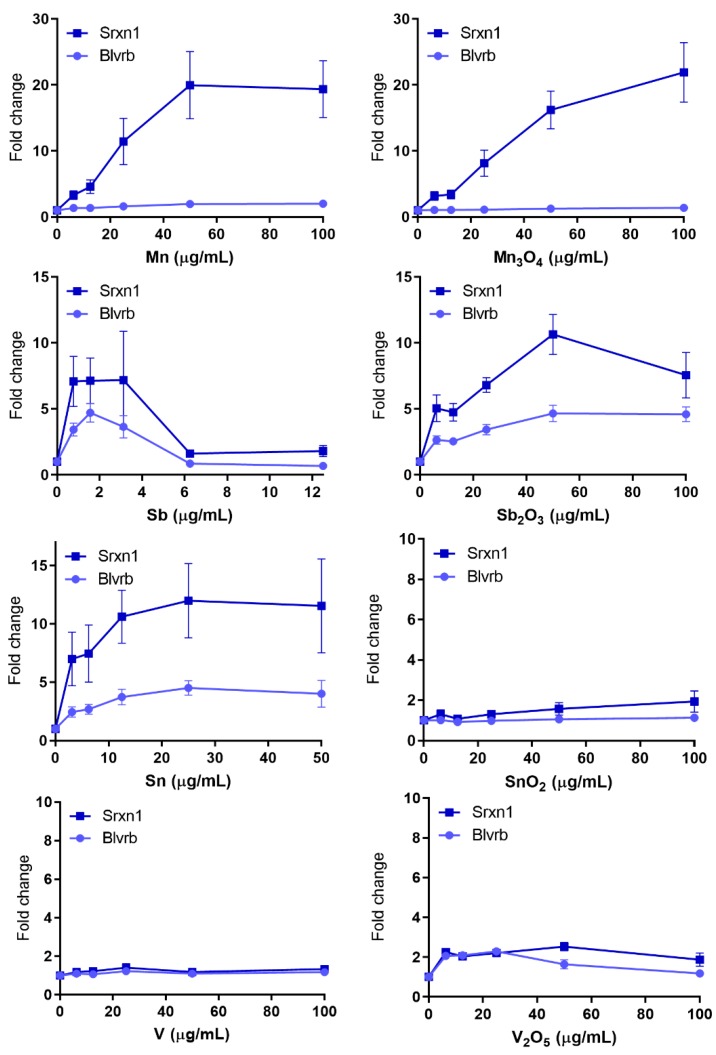
Oxidative stress GFP-reporter activation in response to metal and metal oxide NPs. ToxTracker reporter activation in live cells was measured as GFP-fluorescence using flow cytometry. The Srxn1-GFP reporter is Nrf2-associated, while the Blvrb is Nrf2-independent. The results are presented as mean ± standard error of the mean of three or four independent experiments (*n* = 3–4).

Several of the metal-containing NPs investigated did not cause any major changes in cell viability at doses up to 80–100 µg/mL at 24 h exposure, including Ag, Au, Cr, Cr_2_O_3_, Pt, (data not shown) SnO_2_ and V (Figure 3). Furthermore, these NPs did not cause activation of any of the six GFP-markers, except for Ag NPs which resulted in a minor increase in one of the oxidative stress markers at the highest dose level (Supplement Table S1 and Figure 4). Furthermore, Cr_2_O_3_ caused a weak activation of one of the oxidative stress reporters (Srxn-1) and one of the DNA damage reporters (Rtkn). The absence of activation of any of the GFP-reporters has previously been observed in response to other NPs including TiO_2_, Fe_3_O_4_ and Co_3_O_4_ [10,12].

### 3.3. Cytotoxicity—Several NPs and QDs Affect Cell Viability

In contrast to the non-cytotoxic NPs, several other NPs caused effects on viability at various doses (Figure 3 and Figure S4). The most toxic NPs were the Sb NPs with a LD50 already at 1.53 µg/mL (Figure 3). This was comparable to the cytotoxic effect induced by the smallest (1.4 nm) QDs (LD50 1.1 µg Cd/mL) (Supplementary Figure S4). The Sb NPs were more cytotoxic compared to the Sb_2_O_3_ NPs (LD50 90 µg/mL). Similarly, the Mn (LD50 86 µg/mL) and the Sn NPs (LD50 29 µg/mL) were more toxic compared to their corresponding oxides (Mn_3_O_4_ and SnO_2_; LD50 > 100 µg/mL). In contrast, the V_2_O_5_ NPs (LD50 31 µg/mL) were more cytotoxic than the metal V NPs (LD50 > 100 µg/mL).

The results on cytotoxicity showed large variances between the differently sized CdTe QDs, demonstrating smaller size of QDs to be toxic at considerably lower doses compared to the larger sized particles (Supplementary Figure S4). The LD50 values for QDs of primary sizes of 1.5, 2.6, 4.5, 6.5 and 8.6 nm were approximately 1, 2, 4, 8.7 and 15.8 µg Cd/mL, respectively. A soluble salt of Cd (CdCl2) was found to be most toxic, with an LD50 of 0.69 µg Cd/mL.

### 3.4. Oxidative Stress—Many NPs and All QDs Induce Reporters Related to Oxidative Stress

The most frequently activated reporter was the Srxn1-GFP reporter. The Srxn1 protein is involved in the reduction of hyperoxidized peroxiredoxin and regulated by the nuclear factor (erythroid derived 2)- like 2 (NRF2) transcription factor, which plays a key role in the oxidative stress response and regulation of various antioxidant gene networks [8,9]. Of the NPs tested, Mn and Mn_3_O_4_ NPs caused by far the largest activation of the Srxn1-GFP reporter resulting in around 20-fold maximum increase compared with control (Figure 4). The Sn, Sb_2_O_3_ and Sb NPs were also active, inducing approximately a 7–10 fold increase (Figure 4), which is comparable to previous observations for Ni and NiO NPs [11], and for Co and CoO NPs [12]. Some induction (2.5 and 2.7-fold) was also observed for the V_2_O_5_ and Ag NPs, respectively (Figure 4 and Supplementary Table S1).

**Figure 5:**
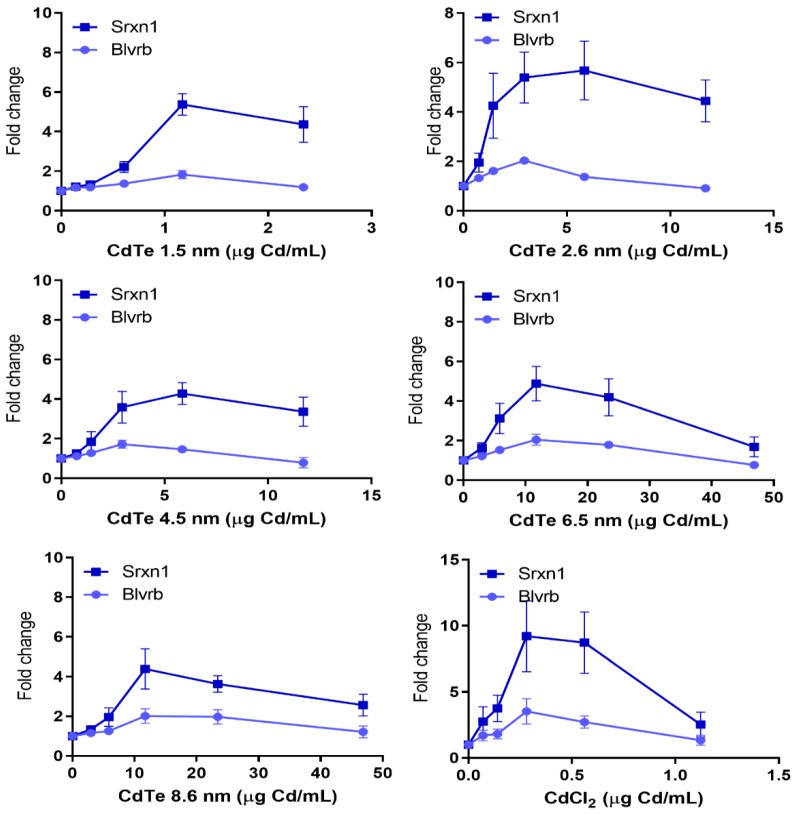
Oxidative stress GFP-reporter activation in response to CdTe QDs of various sizes and CdCl_2_. ToxTracker reporter activation in live cells was measured as GFP-fluorescence using flow cytometry. The Srxn1-GFP reporter is Nrf2-associated, while the Blvrb is Nrf2-independent. The results are presented as mean ± standard error of the mean of three or four independent experiments (*n* = 3–4).

A strong induction of the oxidative stress related Srxn1-GFP reporter was observed for all QDs (Figure 5). The induction started at similarly low doses for the 3 smallest sizes and at somewhat higher doses for slightly larger particles sized 6.5 and 8.6 nm. CdTe QDs of 1.5 and 2.6 nm seemed to be most potent reaching up to a 5-fold increase compared to control. An increase was also observed for Srxn1 in response to the soluble CdCl_2_ salt starting at a lower dose of Cd compared to the QDs and reaching a maximum of 9-fold increase.

Fewer NPs activated the second oxidative stress marker Blvrb. This marker has been shown to play an important role in heme metabolism and has been associated with cellular antioxidant response. However it does not contain any NRF2 binding motif and thus does not overlap with the signaling pathways associated with Srxn1-GFP reporter [8]. Exposure to the Sn, Sb_2_O_3_, Sb and Mn NPs resulted in a maximum increase of approximately 4.5, 4.5, 3.5 and 2-fold, respectively (Figure 4). In addition, a weaker yet positive (or borderline) induction was observed for the QDs of sizes 2.6, 6.5 and 8.6 nm while the soluble CdCl_2_ salt resulted in a 3.5-fold increase (Figure 5). None of the previously studied NPs has been found to activate this marker [11,12,13].

### 3.5. DNA Damage—Few NPs Induce Reporters Related to DNA Damage

**Figure 6:**
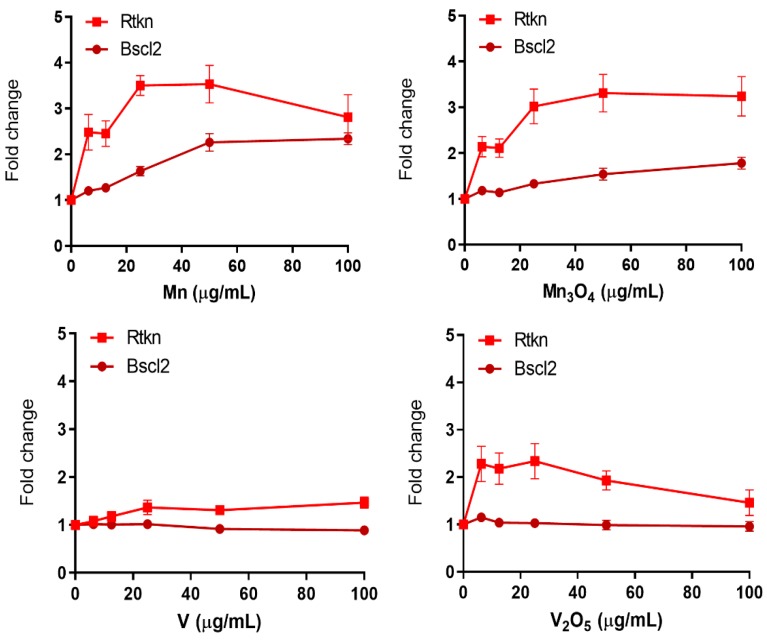
DNA damage GFP-reporter activation in response to metal and metal oxide NPs. ToxTracker reporter activation in live cells was measured as GFP-fluorescence using flow cytometry. The Bscl2-GFP and Rtkn-GFP report on DNA damage associated with ATR/Chk1 or NF- kB signaling, respectively. The results are presented as mean ± standard error of the mean of three or four independent experiments (*n* = 3–4).

In general, few NPs induced reporters related to DNA damage (Figure 6). The Rtkn-GFP marker is associated with the NF-Kb cytokine signaling pathway, which further has been associated with activation of the ATM DNA damage kinase [8]. ATM is rapidly activated upon induction of DNA-double strand breaks and directly phosphorylates both CHK2 checkpoint kinase and p53 tumor suppressor resulting in inhibition of cell cycle progression, activation of DNA repair or inducing apoptosis. The Mn and Mn_3_O_4_ NPs were found to induce Rtkn at all doses tested (6.25–100 µg/mL) with a maximum increase corresponding to 3.5 and 3.3-fold, respectively (Figure 6). A marginal increase was also observed in response to V_2_O_5_ NPs, although at rather cytotoxic doses (40–50%), whereas no induction was observed for the V NPs (Figure 6). In our previous studies, CuO (unpublished), Co, and CoO NPs [12] as well as welding fume NPs [13] have activated the Rtkn reporter at magnitudes ranging from 2 to 3-fold increase. A slight increase (above 1.5-fold) was also observed for Cr_2_O_3_ NPs, as also previously observed for Ni and NiO NPs [11].

The second DNA damage reporter Bscl2 is associated with DNA replication and activation of the ATR (ataxia telangiectasia and Rad3-related) DNA damage signalling pathway [9]. Exposure to the Mn NPs resulted in an activation of the Bscl2 just above the 2-fold threshold (Figure 6). As of now, none of the other NPs investigated in this or previous studies, in total 32 different NPs [10,11,12,13] has clearly induced the Bscl2 reporter. This indicates that most likely none of the tested NPs, or released metal species, could bind directly to DNA and cause stalled replication forks.

None of the QDs or CdCl_2_ had an effect on the DNA damage markers Rtkn or Bscl2 (Supplementary Table S1), except for a borderline increase of Rtkn in response to the 2.6 nm sized QDs.

### 3.6. Protein Unfolding and p53 Related Stress—Few NPs and QDs Induce These Reporters

**Figure 7:**
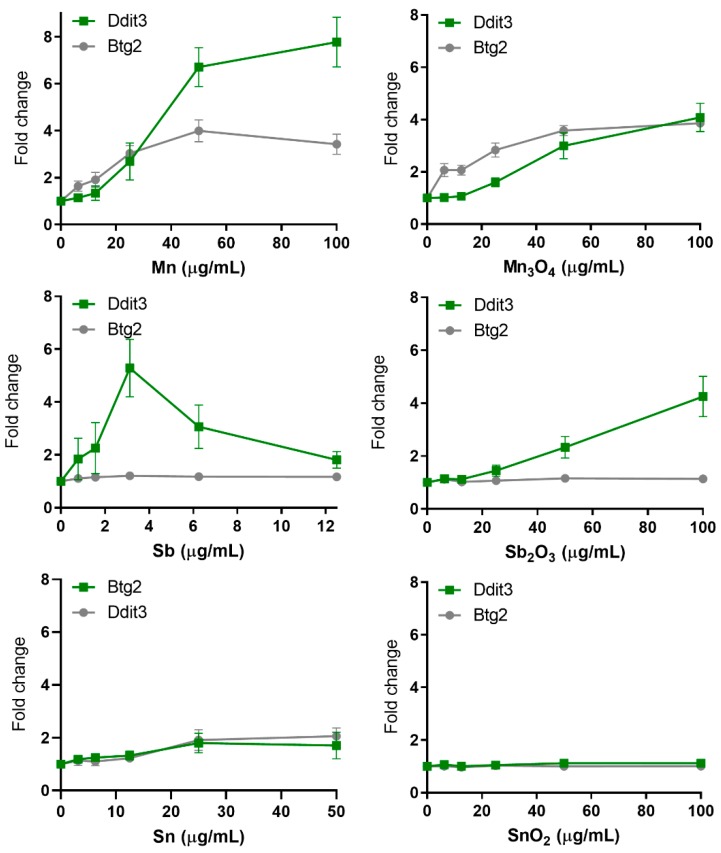
Protein and general cellular stress GFP-reporter activation in response to metal and metal oxide NPs. ToxTracker reporter activation in live cells was measured as GFP-fluorescence using flow cytometry. The Ddit3-GFP and Btg2-GFP indicate unfolded protein response or p53-associated cellular stress, respectively. The results are presented as mean ± standard error of the mean of three or four independent experiments (*n* = 3–4).

The unfolded protein response (UPR) is an additional pathway associated with carcinogenicity. Ddit3 (DNA damage-inducible transcript 3) is a transcriptional factor associated with multiple functions such as cell cycle arrest, apoptosis and endoplasmic reticulum (ER) stress. It further encodes the transcription factor CHOP, which has a vital role in the response to a wide variety of cell stressors following ER stress [8]. The Mn NPs revealed the strongest induction of the Ddit3-GFP reporter, with a maximum of almost 8-fold increase (Figure 7). This is equivalent to the maximum fold induction of Ddit3-GFP historically observed for ToxTracker [8]. This high increase was followed by the Mn_3_O_4_ and Sb_2_O_3_ NPs, both inducing a 4-fold increase, as well as the Sn NPs with an increase marginally exceeding the 2-fold threshold (Figure 7). No increase was observed in response to the SnO_2_ NPs (Figure 7). Exposure to Sb NPs further resulted in an increase of the Ddit3-GFP reporter, reaching a 2 to 5-fold increase, although results exceeding the 2-fold level were observed at viability levels less than 25% (Figure 7).

**Figure 8:**
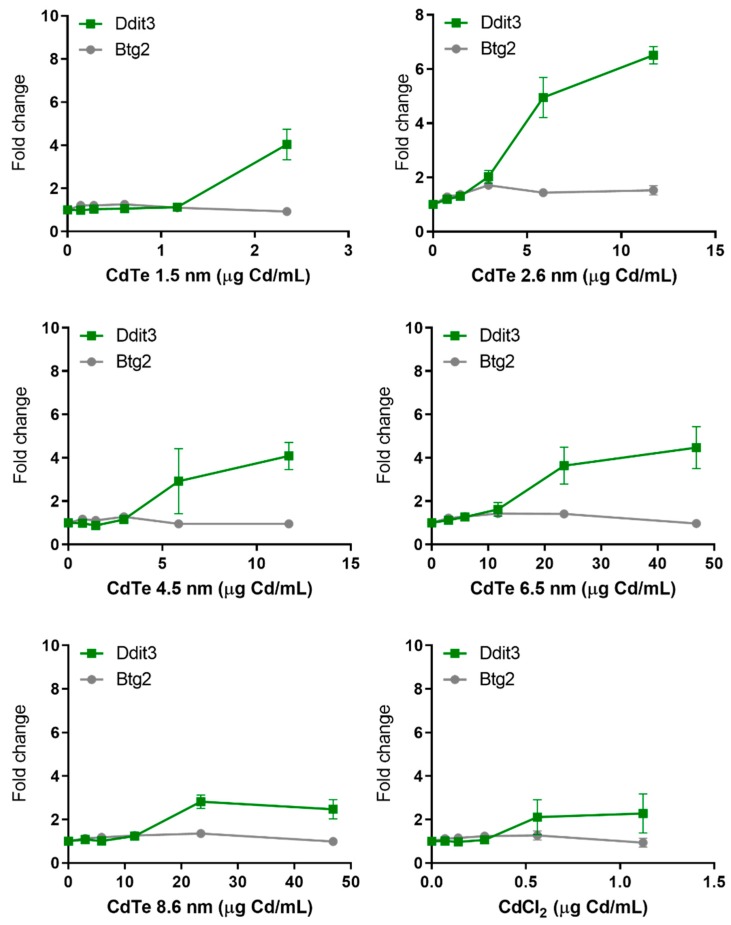
Protein and general cellular stress GFP-reporter activation in response to CdTe QDs of various sizes and CdCl_2_. ToxTracker reporter activation in live cells was measured as GFP-fluorescence using flow cytometry. The Ddit3-GFP and Btg2-GFP indicate unfolded protein response or p53-associated cellular stress, respectively. The results are presented as mean ± standard error of the mean of three or four independent experiments (*n* = 3–4).

A clear induction of the protein stress UPR-marker Ddit3 was observed for all QDs, where the induction started at lower doses for the 3 smallest sized QDs compared to the larger sized ones (Figure 8). CdTe QDs sized 2.6 nm was the most potent, inducing up to 6.5-fold increase, although this was observed at a viability level less than 25%. Noteworthy is that the QDs sized 1.5 and 6.5 nm only resulted in an induction of Ddit3 at a viability level less than 25%. The soluble CdCl_2_ salt caused an approximately 2-fold increase.

In general, very few of the investigated NPs resulted in an activation of the p53-dependent Btg2-GFP reporter. Only the Mn and Mn_3_O_4_ NPs induced the reporter with approximately a 4-fold increase (Figure 7). No effect was observed on the p53-responsive Btg2-GFP reporter for either QDs or soluble CdCl_2_ (Figure 8). These findings can be compared to previous studies, where welding fume NPs were the only NPs that resulted in an activation at comparable levels, approximately 4-fold [13]. In addition, NiO NPs were seen to induce the Btg2-GFP reporter in the first study by Karlsson et al. [10]. However, the same effect was not observed in a later study by Akerlund et al. [11].

### 3.7. Dose Metric: Surface Area Appears to be the Most Suitable Dose Metric for CdTe QD Toxicity

**Figure 9:**
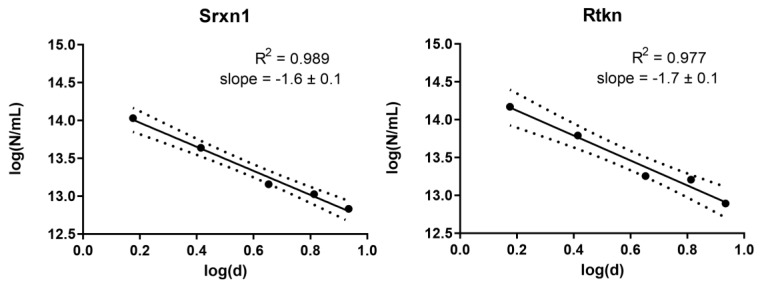
Equi-response curves for CdTe QDs (1.5, 2.6, 4.5, 6.5, 8.6 nm) representing a 20% reduction in cell viability compared to control for Srxn1 and Rtkn reporter. Slopes are plotted according to the model proposed by Delmaar et al. [24] where N denotes the number of particles and d the diameter of the particle.

Since the metal- and metal oxide NPs of this study were not investigated in different sizes, these results cannot be used to further investigate the appropriate dose metric. For the QDs, however, five different particle sizes ranging from 1.5 to 8.6 nm were investigated. To assess the most appropriate dose metric for cytotoxicity induced by the CdTe QDs, we used the approach suggested by Delmaar et al. [24]. Consequently, the equi-response doses representing 20% decrease in cytotoxicity were plotted as a point in the plane spanned by the 10log of the diameter and the 10log of the number of particles (Figure 9 and Supplementary Figure S5). The results showed that the slopes of the calculated curves (ranging between −1.4 to −1.7) were closest to the value of −2 for all reporter cell lines, which suggests that the surface area [24] is the most appropriate dose metric (i.e., the total surface area of NPs causes similar toxicity no matter the size of the NPs).

**Figure 10:**
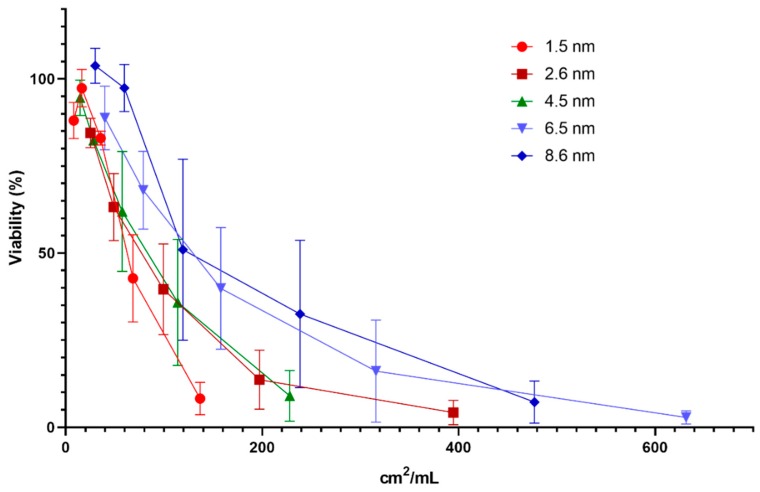
Viability in mES cells following 24 h exposure to CdTe QDs of varying sizes at doses expressed as surface area (cm^2^/mL). Cytotoxicity was determined by measuring the fraction of intact cells following exposure using flow cytometry. The results are presented as mean ± standard error of the mean of three or four independent experiments (*n* = 3–4).

Further, the exposure dose (μg/mL) of the QDs was converted to surface area (cm^2^/mL), based on supplier’s information, which was plotted against viability for all 5 sizes of QDs (Figure 10). The results showed similar and somewhat overlapping curves for the two largest sized particles (8.6 and 6.5 nm), and similarly the curves of the QDs with size of 4.5 and 2.6 nm, respectively, were comparable. The smallest QDs tested resulted in a somewhat steeper curve compared to the larger sizes. These results support the conclusion of surface area being a good dose metric for QDs, but also indicates that the smallest QDs (1.5 nm) could have additional toxic effects not entirely explained by the larger surface area.

## 4. Discussion

The increasing manufacturing and usage of NPs require faster and more efficient testing of their potential toxic effects. Traditional in vitro genotoxicity assays are often time-consuming and have the limitations that they frequently lack the ability to provide mechanistic insight. One alternative approach, as employed in this study, is to assess the cellular stress response pathways that are activated after exposure to NPs using ToxTracker assay. The results from this study show that whereas many NPs induce oxidative stress, few of them induce DNA damage reporters. The NPs with most remarkable response in the ToxTracker assay were the Mn and Mn_3_O_4_ NPs causing massive oxidative stress, induction of reporters for DNA damage, protein unfolding and p53-related stress. This study also highlights the clear particle size dependent toxicity of CdTe QDs.

Our present study supports the general notion that ROS generation and oxidative stress is one of the main mechanisms behind the toxicity of NPs, of which the Mn-containing NPs clearly were the most active. Similar findings have been observed in other studies. Mn_3_O_4_ NPs have been shown to induce the highest ROS level among four different metal oxides tested (Mn_3_O_4_, TiO_2_, Fe_2_O_3_ and Co_3_O_4_) in A549 cells [35] as well as in rat macrophage cell lines [36]. In addition, Mn_3_O_4_ NPs induced the highest amount of acellular ROS out of 11 metal oxides tested [37]. The Mn-based NPs also caused induction of the DNA damage reporter Rtkn. The Rtkn reporter is activated upon induction of DNA double strand breaks and likely, high levels of ROS production caused by the Mn NPs can result in high levels of DNA single strand breaks that in turn will lead to DNA double strand breaks during DNA replication. The Mn NPs were further the only NPs investigated so far (in total 33 different NPs) that caused a clear induction of the DNA damage reporter Bscl2, indicating DNA damage that result in interference of replication. The literature seems scarce on the genotoxic effects of Mn NPs. However, Alarifi et al. [38] reported MnO_2_ NPs to induce DNA damage in human neuronal SH-SY5Y cells. In another study, MnO_2_ NPs were found to cause genotoxicity in vivo with a significant increase in DNA damage in leukocytes as well as micronuclei and chromosomal aberrations in bone marrow cells after oral exposure [39]. Based on the ToxTracker reporter response in this study, it appears somewhat surprising that the Mn-based NPs only showed minor cytotoxicity.

Another response clearly observed for the Mn-based NPs was the protein unfolding response (Ddit3-reporter) related to ER stress. As stated in the review by Cao et al. [40], ER stress has been proposed as a possible mechanism for NP induced toxicity and several metal NPs have been found to induce ER stress in various test systems in vitro such as Ag [41], Au [42], SiO_2_ [43] and ZnO NPs [44]. In our study, we observed this effect only for the Mn and Mn_3_O_4_ NPs as well as for the QDs. Mn ionic species (from MnCl_2_) have previously been shown to induce ER stress in neuronal cells that is proposed to be closely linked to their neurodegenerative effect [45].

Five QDs of different size were investigated in the present study allowing for exploration of size-dependent effects. The cytotoxicity observed after exposure to the six different reporter cell lines clearly demonstrate that QDs of smaller size are more cytotoxic compared to their larger sizes. Our calculations suggest that surface area is a more appropriate dose metric for the QDs measured, while the results also indicate that the smallest sized QDs may have additional toxic effects other than that explained by increased surface area. This is in line with previous studies reporting the same size dependent trend in BEAS-2B [46], L929 mouse fibroblasts [47] and Rat pheochromocytoma PC12 cells [20]. The generation of ROS and oxidative stress in response to CdTe QDs has previously been shown in various cell types [21,47,48,49]. Furthermore, the addition of the antioxidant NAC has been reported to reduce CdTe QD cytotoxicity and preserve mitochondrial morphology [20,21]. The activation of UPR observed in response to CdTe QDs in this study is in line with findings of Jiang et al. [50], demonstrating low concentrations of CdTe QDs to be distributed at ER, resulting in ER expansion and UPR activation in HEK kidney cells. These findings were further confirmed in the kidneys of mice exposed to CdTe QDs, demonstrating that UPR mediates the toxicity of CdTe QDs both in vitro and in vivo [50]. CdTe QDs have also been shown to target endothelial ER in human umbilical vein endothelial cells (HUVECs) by causing ER stress, activating the UPR pathway and all of the three downstream ER stress-mediated apoptosis pathways, and ultimately triggered ER stress-induced apoptotic cell death [51].

When comparing effects observed in ToxTracker between the QDs and the soluble CdCl_2_ salt, based on Cd-content, CdCl_2_ induced cytotoxicity at a lower concentration of Cd compared to that of CdTe QDs sized 1.5 nm. This is somewhat in conflict with previous findings demonstrating CdTe QDs to be more cytotoxic compared to CdCl_2_ at similar concentrations of Cd in HepG2 [52] and HEK cells [50]. Cho et al. [53] report no dose-dependent correlation between cell viability and intracellular [Cd^2+^] following exposure to CdTe QDs, implying the cytotoxicity observed in MCF-7 cell not solely to be related to free Cd ions or Cd-complexes in solution. In the present study, CdCl_2_ was further found to induce the Srxn1 as well as the Ddit3-GFP markers at a lower dose and greater magnitude compared to the smallest sized CdTe QDs (1.5 nm).

In this study, several metallic NPs were compared with their corresponding bulk oxides. Mn- based NPs (Mn, Mn_3_O_4_) were shown to have a very similar potency indicating that they may be grouped together for risk assessment. This may be related to the similar composition of the outermost surface for the metal and the oxide, the actual interface between the particles and the cells, and highlights its importance for the cytotoxic potency. In both cases of the Sb- and Sn-based NPs, the metallic form (metal core with surface oxides) was found to be more toxic and reactive compared to their corresponding bulk oxides (Sb_2_O_3_ and SnO_2_), despite the fact that these oxide NPs had a lower primary size (Figure 1). Sedimentation of the Sb NPs was faster compared with its corresponding oxide (Sb_2_O_3_ NPs, Figure 2), which resulted in a higher cellular dose that may influence the toxicity. This was, however, not the case for the Sn NPs. The V NPs showed rapid sedimentation but were still less toxic than the corresponding oxide (V_2_O_5_ NPs). Thus, it seems difficult to draw any general conclusions regarding the role of surface oxide composition, particle shape/morphology, and sedimentation rate for the toxic potency of metal NPs vs. their corresponding bulk oxide NPs.

One general concern when studying toxicity of NPs is their possible interaction with the different assays, as has been suggested for genotoxicity assays such as comet assay, micronucleus assay and Ames test [54,55,56]. One possible concern could be lack of cell uptake in the mES cells. This is unlikely since we previously showed clear uptake of the NPs [10]. Another problem may be if the NPs emit fluorescence causing a risk of false positive results. By always testing the non-modified mES cells in a dose finding study such an increase in fluorescence can be corrected for, as we did for the QDs in this study. Taken together, assay interaction appears not to be a problem if possible fluorescence of the NPs is corrected for.

**Figure 11:**
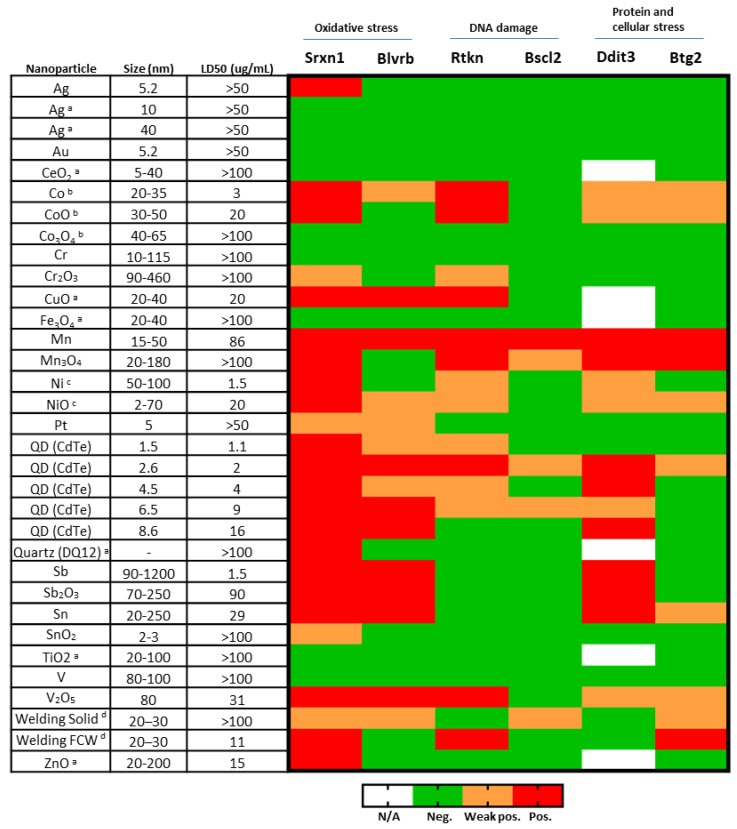
Heatmap of ToxTracker activation of all NPs tested. Size is approximate and based on information from the supplier or TEM imaging. LD50 corresponds to 50% decrease in viability and is based on viability in Srxn1 reporter cells. Weak positive corresponds to >1.5 but <2 fold increase, while positive corresponds to > 2 fold increase at viability levels above 25%. ^a^ Karlsson et al. [10], ^b^ Cappellini et al. [12], ^c^ Akerlund et al. [11], ^d^ McCarrick et al. [13]; welded using solid or flux cored wire, respectively.

Taken together with previous data published on ToxTracker findings, the results on the different NPs (*n* = 33) show a great diversity in activation of GFP-markers as well as in magnitudes of induction, as visualized in Figure 11. This indicates that ToxTracker is a sensitive method for mechanistic screening of NPs and thus able to distinguish differences in toxic effects between various NPs tested. It is evident that the oxidative stress related reporters are the most frequently induced with 20 out of 33 different NPs investigated inducing the Srxn1-GFP reporter. The following NPs clearly induced (exceeding the 2-fold threshold) the Rtkn DNA damage reporter: CuO, Co, CoO, CdTe QDs, Mn, Mn_3_O_4_, V_2_O_5_, and welding NPs. Some induction (>1.5-fold, but less than 2-fold) of the Rtkn reporter was also observed with the Ni, NiO and Cr_2_O_3_ NPs. We suggest that the NPs activating Rtkn reporter are of particular concern when considering genotoxicity of metal- and metal oxide NPs. Ten of the investigated NPs (CdTe QDs, Mn, Mn_3_O_4_, Sb, Sb_2_O_3_ and Sn) also induced the Ddit3-GFP reporter, supporting protein stress as a possible mechanism behind their toxicity.

## 5. Conclusions

In conclusion, we have shown the applicability of using the ToxTracker assay for rapid screening of genotoxic properties of NPs of different size and characteristics, and its suitability as a tool for read-across and for assessing possible toxic mechanisms for further in-depth investigations.

## Figures and Tables

**Table 1:** ToxTracker GFP-reporters.

Biological Damage	Cellular Pathway	Biomarker Gene
Oxidative stress	NRF2 antioxidant response	Srxn1
	NRF2 independent	Blvrb
DNA damage	NF-kB signaling	Rtkn
	ATR/Chk1 DNA damage signaling	Bscl2
Protein damage	Unfolded protein response	Ddit3
Cellular stress	P53 signaling	Btg2

**Table 2:** Summary of primary particle size, approximated particle morphology and main surface oxide composition of the metal and metal oxide NPs based on XRD, Raman and XPS measurements. See Figures S1–S3 and supporting information for surface analytical findings supporting the compositional analysis of the outermost surface oxide.

Particle	Primary Size (TEM)/nm	Oxide Composition
Ag	Spherical, 5.2 ± 1.1 ^a^	N/A
Au	Spherical, 5.2 ± 0.9 ^a^	N/A
CdTe QDs	Spherical, 1.6, 2.6, 4.5, 6.5, 8.6 ^b^	N/A
Cr	Cubic and spherical, 10–115	Cr */Cr_2_O_3_
Cr_2_O_3_	Oval, 90–460	Cr_2_O_3_
Mn	Spherical, 15–50	Mn */MnO/MnO_2_/Mn_2_O_3_/Mn_3_O_4_
Mn_3_O_4_	Cubic 20–180, rods 8000 × 400	Mn_3_O_4_/MnO
Pt	Spherical, 4.8 ± 0.8 ^a^	N/A
Sb	Irregular, 90–1200	Sb */Sb_2_O_3_
Sb_2_O_3_	Spherical, 70–250	Sb_2_O_3_
Sn	Spherical, 20–250	Sn */SnO/SnO_2_
SnO_2_	Spherical, 2–3 (as aggregates)	SnO_2_
V	Rods 20 × 20 to 400 × 40	V_2_O_5_/VO_2_
V_2_O_5_	Irregular 40–450	V_2_O_5_/VO_2_

* metallic signal implies a thin surface oxide, <5–10 nm. N/A not analyzed. ^a^ from Lebedova et al. [23], ^b^ based on supplier information.

## Supplementary Materials

The following are available online at https://www.mdpi.com/2079-4991/10/1/110/s1, Methods for Raman spectroscopy, X-ray photoelectron spectroscopy, X-ray diffraction, Results for oxide characterization including Figure S1: XRD spectra of Mn NPs and Mn_3_O_4_ NPs, Figure S2: XRD results for SN and SnO_2_ NPs, Figure S3: Raman spectra of Sb and Sb_2_O_3_ NPs as well as Figure S4: Viability of CdTe QDs, Figure S5: Equi-response curves for CdTe QDs and Table S1: Maximum ToxTracker activation.
