# Supplementary material for: ToxTracker Reporter Cell Lines as a Tool for Mechanism-Based (Geno)Toxicity Screening of Nanoparticles—Metals, Oxides and Quantum Dots
Source: Nanomaterials (Basel). 2020 Jan 6;10(1):110. doi: 10.3390/nano10010110 (PMC7023144; doi:10.3390/nano10010110)
Supplement: Supplementary file 1 [file nanomaterials-10-00110-s001.pdf]

nanomaterials

Supplementary material

ToxTracker reporter cell lines as a tool for mechanism-based (geno)toxicity screening of nanoparticles – metals, oxides and quantum dots

Sarah McCarrick[1], Francesca Cappellini[1], Amanda Kessler[2], Nynke Moelijker[3], Remco Derr[3], Jonas Hedberg[2], Susanna Wold[2], Eva Blomberg[2, 4], Inger Odnevall Wallinder[2], Giel Hendriks[3], Hanna L Karlsson[1]

[1] Institute of Environmental Medicine, Karolinska Institutet, Stockholm, Sweden.

[2] KTH Royal Institute of Technology, Department of Chemistry, Division of Surface and Corrosion Science, , Stockholm , Sweden.

[3] Toxys, Leiden, The Netherlands.

[4] RISE Research Institutes of Sweden, Division Bioscience and Materials, Stockholm, Sweden

---

Supplementary materials and methods – oxide characterization

Raman spectroscopy

Raman spectroscopy was performed using a Horiba Yvon Jobin HR800 Raman spectrometer. Dry powders were studied using a 785 nm laser and a 50X microscope objective. The powders were checked using an optical microscope for laser beam-induced damages.

X-ray photoelectron spectroscopy

X-ray photoelectron spectroscopy (Kratos AXIS UltraDLD X-ray photoelectron spectrometer, Kratos Analytical, Manchester, UK) studies were performed on dry powder applied onto carbon tape. High-resolution detailed spectra (pass energy 20 eV) were acquired using monochromatic Al Kα X-ray source on Cr 2p, Mn 2p, Sn 3d, Sb 3d, V 4f and O 1s. All binding energies were corrected versus the C 1s adventitious peak at 285.0 eV.

X-ray Diffraction

PANanalytical X'Pert PRO diffractometer (CuKα1λ = 1.54060 Å, CuKα2λ = 1.54443 Å), generator settings at 30mA and 45kV, 2θ range from 25 to 130° with step size 0.01°. Dry powders were studied. Several runs were conducted and averaged to produce spectra with high signal to noise ratio.

Supplementary results - oxide characterization

Manganese (Mn) and manganese oxide (Mn_3O_4) NPs

XRD spectra of Mn and Mn_3O_4 NPs (Figure S1) showing the presence of MnO and Mn metal peaks for the Mn NPs and the diffraction pattern of Mn_3O_4 and MnO for the Mn_3O_4 NPs. The Mn NPs

displayed in addition vibrational Raman modes at 280, 309, 364, 468, and 650 cm^{-1}, and the Mn_3O_4 NPs essentially the same bands. All observed bands indicate Mn_3O_4 [25], though the presence of Mn_2O_3 cannot be excluded due to peak overlap. The Mn NPs have earlier been characterized by means of XPS indicating a surface oxide composed of MnO_2 and Mn_2O_3/Mn_3O_4. The presence of MnO_2 and Mn_2O_3 was supported by cyclic voltammetry (CV) measurements and MnO by means of XRD findings [26].

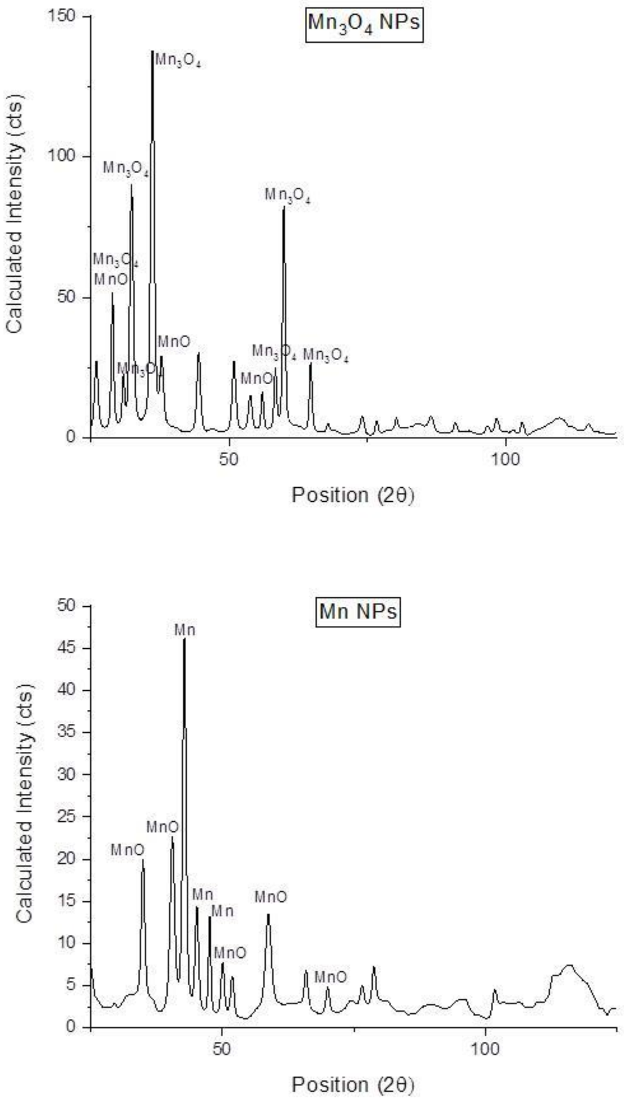

Figure S1. XRD spectra of Mn NPs [3] and Mn_3O_4 NPs (bottom).

Tin [4] and tin oxide (SnO_2) NPs

XRD results for Sn- and SnO_2 NPs are presented in Figure S2. Diffraction peaks corresponding to metallic Sn and the strongest peaks of SnO (minor intensity) were observed for the Sn NPs. The SnO_2 NPs only revealed the main peaks corresponding to SnO_2. The major peaks are marked according to reported assignments [30,31] XPS results imply SnO_2 (binding energy of Sn 3d at 486.4-486.9 eV) [27]

to be a main component of the outermost surface on both the Sn- and the SnO_2 NPs. However, the presence of SnO cannot be excluded due to peak overlap. The metallic signal (484.5 eV) for the Sn NPs implies a thin surface oxide (< 5-10 nm).

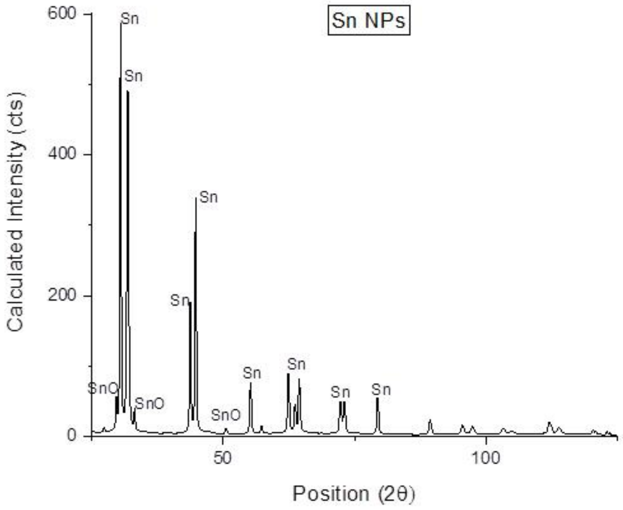

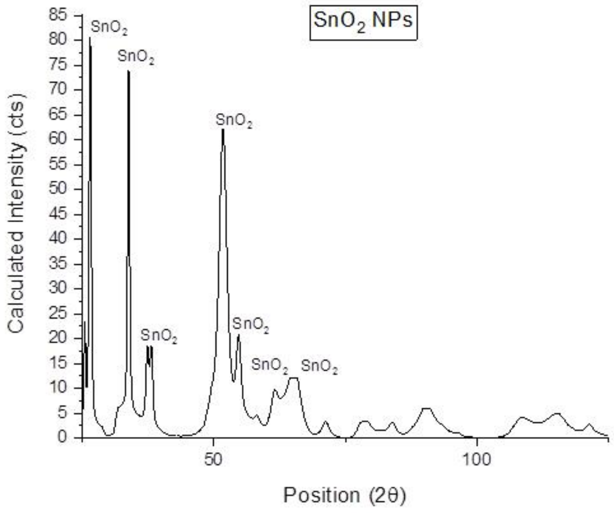

Figure S2. XRD results for Sn NPs [3], and SnO_2 NPs (bottom)**.**

Antimony (Sb) and antimony oxide (Sb_2O_3)

Raman spectra of Sb- and Sb_2O_3 NPs are displayed in Figure S3. The observed Raman bands at approximately 450, 370, 250 and 190 cm-1 correspond to Sb_2O_3 for both NPs [32]. The small difference in peak frequencies of the Sb- and Sb_2O_3 Raman bands may e.g. be attributed to differences in particle size [34] The XPS results imply the presence of Sb_2O_3 on both the Sb- and the Sb_2O_3 NPs, based on observed binding energies at 538.8-539.0 eV [33] The metallic signal observed for the Sb NPs implies a thin surface oxide, < 5-10 nm.

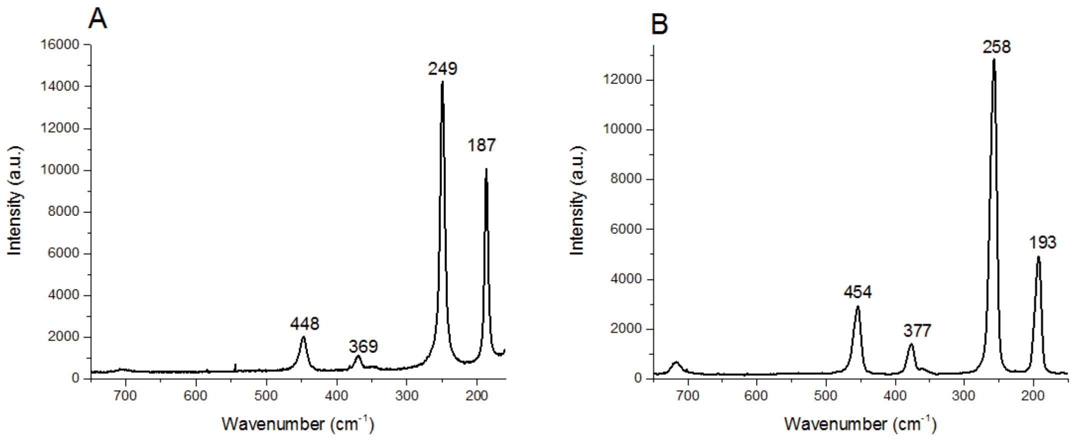

Figure S3. Raman spectra of Sb NPs (A) and Sb_2O_3 NPs (B).

Vanadium (V) and vanadium oxide (V_2O_5) NPs

The V-based NPs were characterized by means of XPS. The results imply the presence of both VO_2 and V_2O_5 oxides in the outermost surface of both the V- and the V_2O_5 NPs (516.2-516.4 eV and 517.4-517.9 eV, respectively [28]. The lack of metal signal of the V NPs indicates a surface oxide thicker than 5-10 nm.

Chromium (Cr) and chromium oxide (Cr_2O_3) NPs

XPS results indicate the presence of Cr_2O_3 in the surface oxide for both Cr and Cr_2O_3 NPs, as judged from the Cr 2p peaks at 578-581.5 eV [29]. The Cr NPs showed in addition also metallic Cr signal (binding energy 574.8 eV) that implies a thin surface oxide, < 5-10 nm [29].

Supplementary results – Viability of CdTe QDs

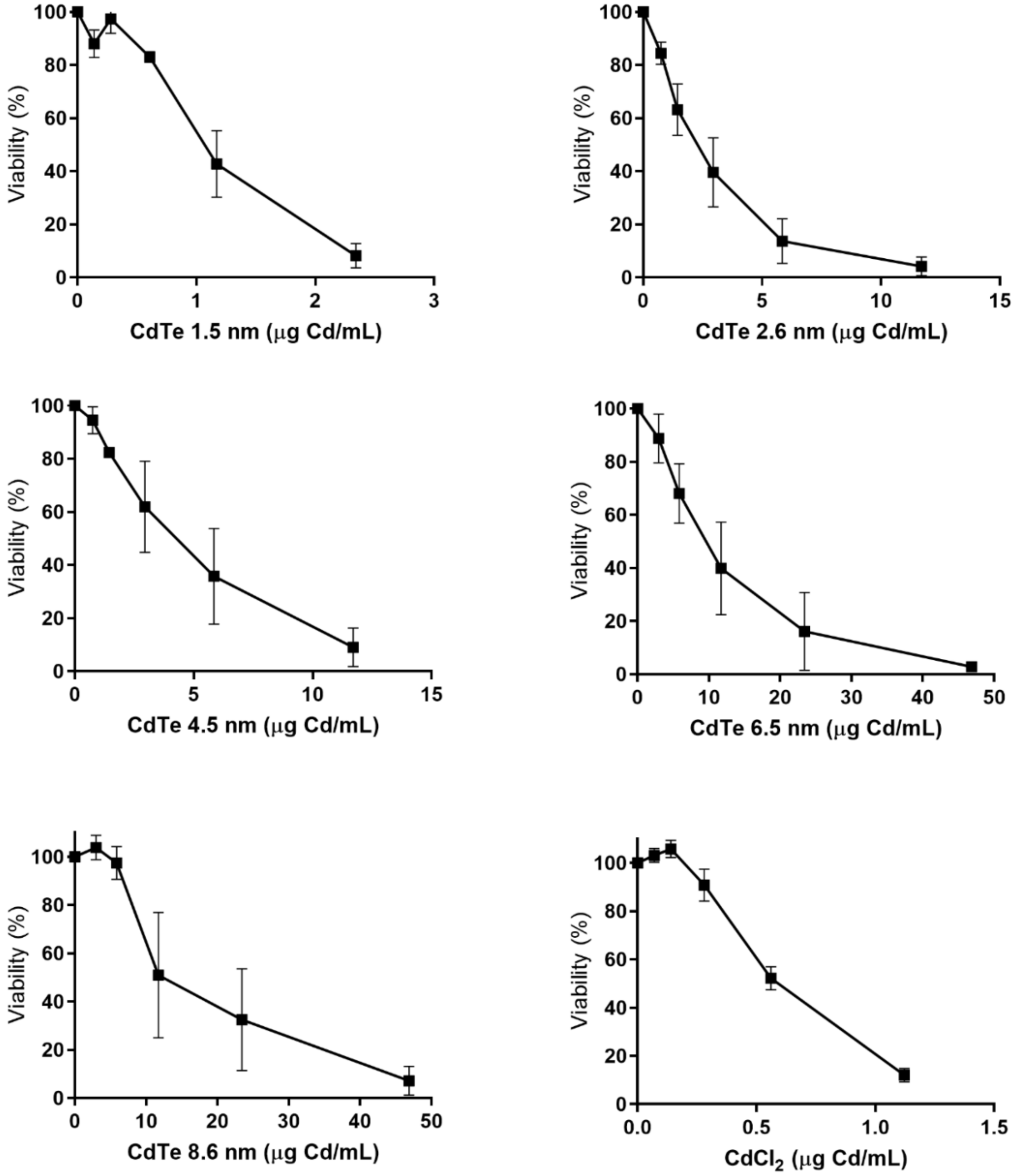

Figure S4. Cytotoxicity in mES cells following 24 h exposure to CdTe QDs of 5 different sizes as well as $CdCl_2$. Cytotoxicity was determined by measuring the fraction of intact cells following exposure using flow cytometry. The results are presented as mean ± standard error of the mean of three or four independent experiments (n=3-4).

Supplementary results – Maximum ToxTracker activation

Table S1. Maximum ToxTracker activation observed in response to metal and metal oxide NPs as well as CdTe QDs of various sizes and $CdCl_2$. Values correspond to maximun ToxTracker activation at viability levels above 25 %. Asterisks (*) indicate an induction observed at a viability level below 50 %.

NP/ compound	Oxidative stress		DNA damage		Cellular stress	
	Srxn	Blvrb	Rtkn	Bscl2	Ddit3	Btg
**Ag**	2.67 ± 0.29	1.49 ± 0.09	1.06 ± 0.05	1.04 ± 0.03	1.15 ± 0.03	0.98 ± 0.03
**Au**	0.96 ± 0.02	0.99 ± 0.01	0.96 ± 0.03	1.02 ± 0.02	1.03 ± 0.02	0.99 ± 0.01
**$CdCl_2$**	9.21 ± 2.68	3.53 ± 0.96	1.06 ± 0.18	1.19 ± 0.09	2.11 ± 0.9	1.27 ± 0.21
**CdTeQDs 1.5**	5.37 ± 0.54*	1.83 ± 0.2*	1.64 ± 0.29	1.28 ± 0.03	1.13 ± 0.09	1.26 ± 0.08
**CdTeQDs 2.6**	5.39 ± 1.03*	2.03 ± 0.15*	2.06 ± 0.21	1.54 ± 0.04*	2.02 ± 0.24*	1.71 ± 0.07
**CdTeQDs 4.5**	4.28 ± 0.55*	1.72 ± 0.2	1.51 ± 0.14	1.26 ± 0.13	2.92 ± 1.5*	1.28 ± 0.07
**CdTeQDs 6.5**	4.88 ± 0.88*	2.05 ± 0.28*	1.65 ± 0.14	1.53 ± 0.03*	1.62 ± 0.32*	1.43 ± 0.07
**CdTeQDs8.6**	4.39 ± 1.01	2.01 ± 0.36	1.28 ± 0.09	1.29 ± 0.14*	2.82 ± 0.31*	1.35 ± 0.04
**Cr**	1.07 ± 0.16	1.08 ± 0.07	1 ± 0.08	1 ± 0.04	1.03 ± 0.13	0.92 ± 0.07
**Cr_2O_3**	1.71 ± 0.26	1.44 ± 0.08	1.69 ± 0.18	1.06 ± 0.04	1.04 ± 0.06	1.18 ± 0.04
**Mn**	19.96 ± 5.11	2.02 ± 0.13	3.53 ± 0.41	2.34 ± 0.13	7.77 ± 1.06	3.99 ± 0.47
**Mn_3O_4**	21.91 ± 4.51	1.39 ± 0.12	3.31 ± 0.41	1.78 ± 0.13	4.08 ± 0.54	3.86 ± 0.32
**Pt**	1.78 ± 0.05	1.53 ± 0.07	0.96 ± 0.03	1.12 ± 0.02	0.95 ± 0.04	1.01 ± 0.03
**Sb**	7.13 ± 1.73*	4.71 ± 0.71	0.88 ± 0.05	1.23 ± 0.03	5.28 ± 1.09*	1.21 ± 0.13
**Sb_2O_3**	10.64 ± 1.51	4.66 ± 0.63	0.91 ± 0.03	1.19 ± 0.03	4.25 ± 0.76*	1.16 ± 0.12
**Sn**	11.98 ± 3.19*	4.5 ± 0.62*	1.32 ± 0.41	1.24 ± 0.12	2.06 ± 0.32*	1.8 ± 0.37
**SnO_2**	1.94 ± 0.53	1.13 ± 0.11	1.05 ± 0.06	1.04 ± 0.04	1.12 ± 0.08	1.03 ± 0.03
**V**	1.34 ±0.1	1.23 ± 0.07	1.47 ± 0.12	1.02 ± 0.08	0.94 ± 0.16	1.14 ± 0.09
**V_2O_5**	2.53 ± 0.21*	2.29 ± 0.11	2.34 ± 0.37	1.15 ± 0.08	1.55 ± 0.09	1.85 ± 0.16

Supplementary results – dose metric modelling analysis

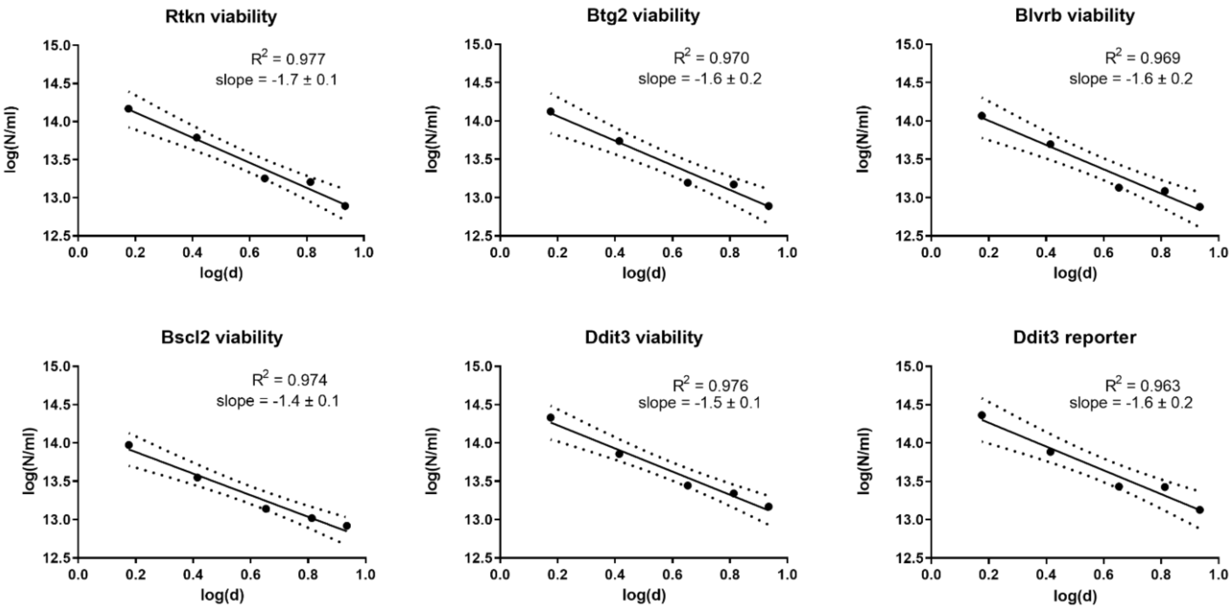

Figure S5. Equi-response curves for CdTe QDs (1.5, 2.6, 4.5, 6.5, 8.6 nm) representing a 20 % decrease in cell viability compared to control or a 1.5-fold increase in reporter activation. Slopes plotted according to Delmaar et al. [24] where Numb stands for number of particles and d stands for diameter of the particle (nm).
